# Supplementary material for: The Human Extracellular Matrix Diseasome Reveals Genotype–Phenotype Associations with Clinical Implications for Age-Related Diseases
Source: Biomedicines. 2023 Apr 19;11(4):1212. doi: 10.3390/biomedicines11041212 (PMC10135578; doi:10.3390/biomedicines11041212)
Supplement: Supplementary file 1 [file biomedicines-11-01212-s001.zip › biomedicines-2303489-figure S1.pdf]

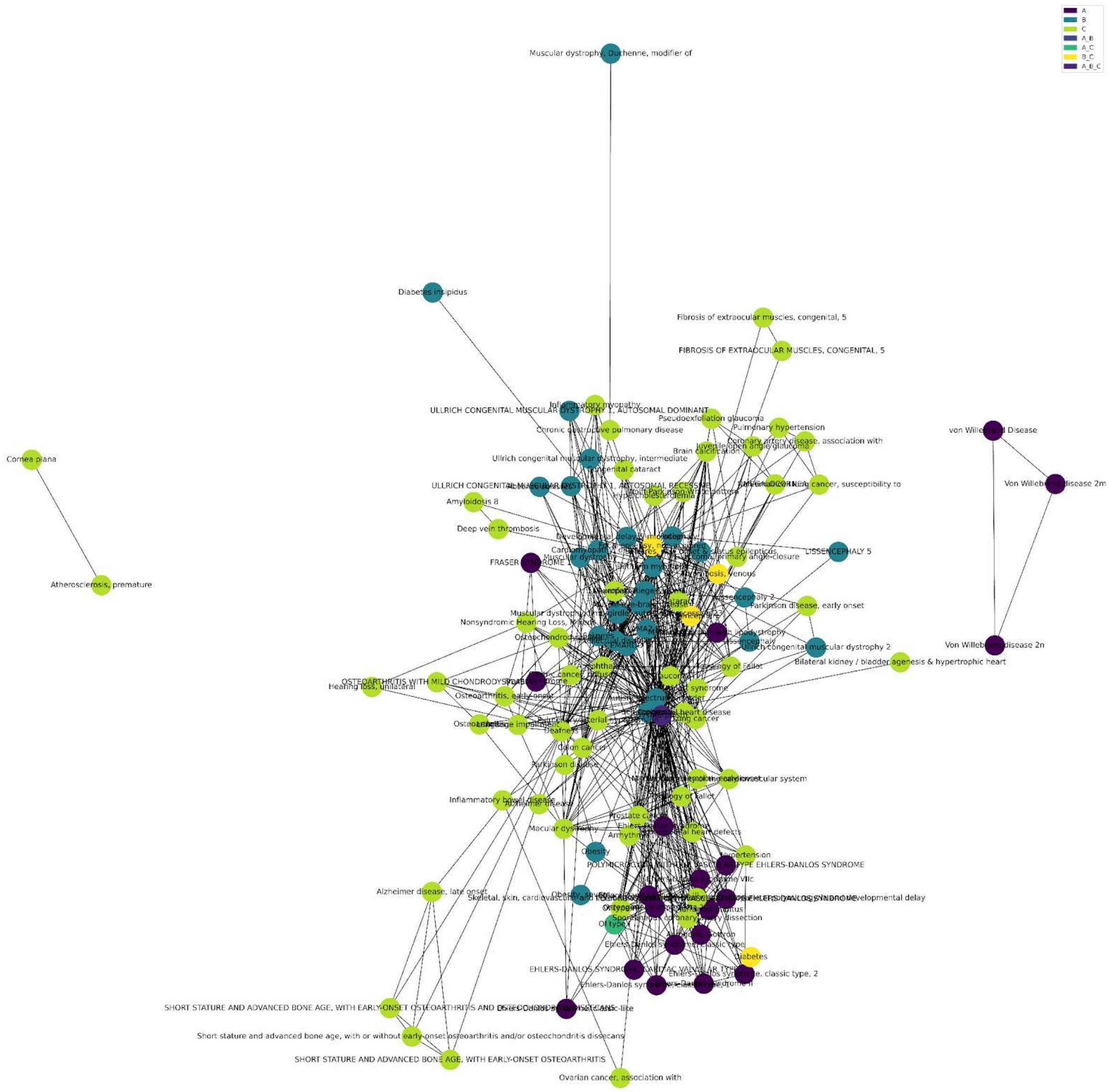

Supplementary Figure 1. Core Matrisome Gene-Disease Associations. Cluster analysis of core matrisome gene-disease associations, where age-related diseases form the predominant category of matrisome and specifically core matrisome gene-disease associations (Supplementary Table 9).
